# Supplementary material for: Real World Estimate of Vaccination Protection in Individuals Hospitalized for COVID-19
Source: Vaccines (Basel). 2022 Apr 1;10(4):550. doi: 10.3390/vaccines10040550 (PMC9031136; doi:10.3390/vaccines10040550)
Supplement: Supplementary file 1 [file vaccines-10-00550-s001.zip › vaccines-1645663-supplementary.pdf]

Supplementary Material

Supplementary Table S1. Relative hazards of in hospital death (primary endpoint) from fitting a standard Cox regression model, unadjusted and adjusted for age, ethnicity, month of enrolment and immunocompetence.

	Unadjusted and adjusted relative hazards of in hospital death			
	**Unadjusted HR (95% CI)**	**p-value**	**Adjusted * HR (95% CI)**	**p-value**
Unvaccinated	1		1	
One dose	1.25 (0.76, 2.03)	0.377	0.96 (0.59, 1.58)	0.886
2+ doses	0.96 (0.65, 1.40)	0.825	0.48 (0.29, 0.82)	0.007

** adjusted for age, ethnicity, immunocompetence and month of enrolment.*

Supplementary Table S2. Relative hazards of in hospital death (primary endpoint) from fitting a standard Cox regression model including only patients with at least two-doses with months from second dose to hospital admission as exposure.

	Unadjusted and adjusted relative hazards of in hospital death			
	**Unadjusted HR (95% CI)**	**p-value**	**Adjusted * HR (95% CI)**	**p-value**
	1		1	
**Time elapsed from second dose per month longer**	1.15 (0.89, 1.47)	0.283	0.88 (0.56, 1.38)	0.572

* adjusted for age, ethnicity, age-unadjusted CCI and month of enrolment.

Supplementary Table S3. Unadjusted and adjusted HR of death from fitting a standard Cox regression model - including only those who initiated cycle with BNT162b2.

	Unadjusted and adjusted relative hazards of in hospital death -subset of those initiating vaccine cycle with BNT162b2			
	**Unadjusted HR (95% CI)**	**p-value**	**Adjusted * HR (95% CI)**	**p-value**
Unvaccinated	1		1	
One dose	2.05 (1.22, 3.43)	0.006	1.50 (0.89, 2.53)	0.128
2+ doses	1.12 (0.75, 1.69)	0.578	0.53 (0.31, 0.90)	0.020

** adjusted for age, ethnicity, age-unadjusted CCI and month of enrolment.*

Supplementary Table S4. Unadjusted and adjusted HR of death from fitting a standard Cox regression model - primary endpoint sensitivity analysis restricted to those with PaO2/FiO2 less than 300 mm/hg.

	Unadjusted and adjusted relative hazards of in hospital death -subset of those with PaO2/FiO2<=300 mm/hg			
	**Unadjusted HR (95% CI)**	**p-value**	**Adjusted * HR (95% CI)**	**p-value**
Unvaccinated	1		1	
One dose	1.37 (0.72, 2.61)	0.344	1.16 (0.60, 2.26)	0.659
2+ doses	1.01 (0.63, 1.61)	0.969	0.57 (0.31, 1.07)	0.081

** adjusted for age, ethnicity, age-unadjusted CCI and month of enrolment.*

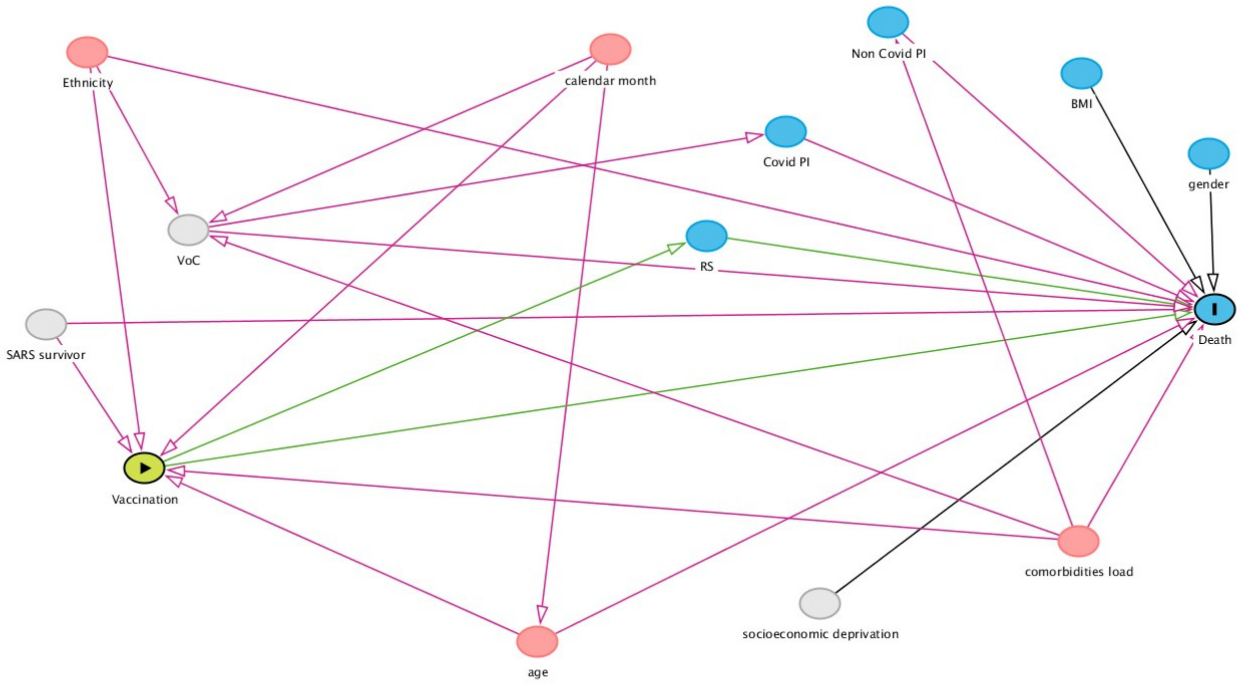

Supplementary Figure S1. Underlying assumptions regarding the causal links between variables.
